# Primitive Practitioners

**Published:** 1891-11

**Authors:** 


### PRIMITIVE PRACTITIONERS.

It is said that Asclepiades, the ancient quack, perambulated the world on a cow's back, living on her milk as he went along. We have no assurance that this mode of conveyance ever became popular with the profession. It was customary, however, for the English in Charles II's time, to visit their patients on horseback, sitting sideways on foot cloths like women. Andery says that Harvey, the great English anatomist, rode on horseback with a foot cloth, his men following on foot, as the fashion then was, which was very decent. Later, carriages of various kinds, some very showy, came into vogue, and until very recently, physicians were distinguished in American cities by the use of a special kind of phæton. It would be profitable to consider the advantages of the bicycle as a hygienic and curative measure for the doctor's patients, but at present we wish to confine our attention to the use of the wheel as a mode of locomotion for the physician himself. This is from the *Medical Gazette*, which in continuing says: A fair horse costs $200 ; buggy, harness, etc., $300 ; year's board, $200 ; shoeing and repairs, $50 ; while the risk of sickness, accident and death are great. For all those provoking calls that come just after one's horse has been sent to the stable, night calls, calls too long to walk, calls too short to drive, the bicycle is always ready and can be depended upon every time to "get there" without delay.

